# Is it possible to use research to learn psychiatry from scratch: a reflective self-study of a pre-clinical year medical student

**DOI:** 10.1192/bjo.2021.436

**Published:** 2021-06-18

**Authors:** Jashan Selvakumar, Jiann Lin Loo, Mary Honey Ohn, Gabby Kelly

**Affiliations:** 1 St Georges University of London; 2 Betsi Cadwaladr University Health Board; 3 University Hospital Lewisham; 4 Cardiff University

## Abstract

**Aims:**

Despite the abundance of opportunities available for medical students to explore the field of psychiatry, active immersion through experiential learning has proven to be difficult for pre-clinical year students as a result of a busy time table and the need to wait for psychiatry postings during the clinical years. Hence, the question of “how to implement experiential learning of psychiatry in pre-clinical years” arises. This study is aimed to elucidate the attempts that have been made to use research as a proximate approach to learn psychiatry experientially, focusing specifically on the challenges faced and lessons learned by a pre-clinical medical student.

**Method:**

This self-study outlined the informal three-months learning-by-doing journey of a year-one medical student, supervised by a psychiatrist registrar. Employing research as a proximate approach of experiential learning for psychiatry was explored based on reflection from discussion during supervision meetings and messages exchange. The agreed learning method was an active involvement in research projects on psychiatry topics, with the learning outcome of producing publications.

**Result:**

The challenges faced included: 1) the difficulty associated with striking a balance between an ambitious project with high impact versus a feasible smaller project to keep both parties motivated through the means of short-term accomplishment; 2) the ongoing requirement for learning process adjustment to build the foundational knowledge essential for progress. Through active and deliberate effort, every step in the process was found to be an opportunity for active learning. Literature review, for example, was used to build the understanding of psychiatry topics and practise critical appraisal skills, while allowing for the recognition of knowledge gaps, which ultimately encouraged future research idea synthesis. The process of writing and submitting a manuscript was used to learn publication-relevant skills including: journal impact calculation, referencing, indexing and abstracting services, and publication ethics. Certain future proof skills were also developed, including literacy in information and communication technology which improved efficiency of research, problem solving and decision making. This was done using pros and cons whenever difficulties were faced.

**Conclusion:**

Although research is not a comprehensive substitute for clinical posting in the process of learning psychiatry, the lessons learned from psychiatry research can potentially serve as an initial exploration tool for preclinical-year medical students interested in the field. The stimulating process has found to be effective in stimulating further interest in psychiatry but maintaining it will be the next challenge.

